# The regulatory network among CircHIPK3, LncGAS5, and miR-495 promotes Th2 differentiation in allergic rhinitis

**DOI:** 10.1038/s41419-020-2394-3

**Published:** 2020-04-02

**Authors:** Xiaoyuan Zhu, Xueping Wang, Ying Wang, Yulin Zhao

**Affiliations:** Department of Rhinology, The First Affiliated Hospital of Zhengzhou University, Zhengzhou, 450052 China

**Keywords:** Cell biology, Chemical biology

## Abstract

Allergic rhinitis (AR) is a common allergic disease which is characterized by the promotion of Th2 differentiation of CD4^+^ T cells. However, the mechanisms underlying Th2 differentiation remain unclear. Non-coding RNAs play a critical role in Th2 differentiation, whereas few studies have revealed the interactions among long non-coding RNAs, circular RNAs, and microRNAs. In this study, the differential expressions of several circRNAs and lncRNAs were compared in nasal mucosa samples of AR patients and mice with experimentally induced AR as compared to healthy controls. The results showed that the highly expressed CircHIPK3 and LncGAS5 promoted Th2 differentiation of ovalbumin-induced CD4^+^ T cells and aggravated nasal symptoms of AR mice. We also found that CircHIPK3 and LncGAS5 induced the upregulation of Th2 cell-specific transcript factor GATA-3 via modulating their common target miR-495. Meanwhile, the intranasal administration of CircHIPK3 or LncGAS5 knockdown lentivirus decreased nasal symptoms of AR mice. In conclusion, our findings indicated that the interactions among CircHIPK3, LncGAS5, and miR-495 play a critical role in the regulation of Th2 differentiation in AR.

## Introduction

Allergic rhinitis (AR) is the most common allergic disease that involves a variety of populations worldwide. The immunological basis for AR mainly includes the differentiation from naive lymphocytes into immune regulatory cells, such as helper T cells (Th) 1 and Th2 cells, that exert the pro- or anti-inflammatory functions^1^. Th2 cells are differentiated from the precursor cells which are naive CD4^+^ T cells, and the pro-inflammatory cytokines like IL-4 and IL-5 were mainly secreted by Th2 cells^2^. Under the pathological condition of AR, the percentage of Th2 cells is increased, causing an abnormal immune response^3^. Therefore, the inhibition of Th2 differentiation provides a potential therapeutic strategy of AR. GATA-3, a transcription factor that can be expressed by Th2 cells, plays an important role in Th2 differentiation during immune responses^4^. It is reported that the high expression of GATA-3 in Th2 cells promotes Th2 differentiation, resulting in the release of large amounts of immunoglobulin E (IgE) from B-cells and finally aggravating AR^5^. However, the regulatory pattern of GATA-3 in Th2 differentiation of AR remains unclear.

Non-coding RNAs (ncRNAs) which mainly include circular RNAs (circRNAs), long non-coding RNAs (lncRNAs), and microRNAs (miRNAs) exert their regulatory roles in many biological processes, such as tumorigenesis, fibrogenesis, and also, immune regulation^6,7^. LncRNAs are characterized with a length of over 200 nucleotides, and expressed in both cytoplasm and nucleus^8^. Accumulating studies have revealed that lncRNAs can act as competing endogenous RNAs (ceRNAs) to regulate gene expression by sponging miRNAs from 3′-untranslated regions (3′-UTRs) of targeting mRNAs^9^. CircRNAs are another type of ncRNAs that characterized by covalently closed-loop structures with neither 5′ to 3′ polarity nor a polyadenylated tail^6^. Recently, the ceRNA mechanism has also been reported in circRNAs^10,11^, highlighting a potential interaction among circRNAs, lncRNAs, and miRNAs. A study conducted by Nan et al.^12^ suggested that a lncRNA (named as lncRpa) and a circRNA (named as circRar1) induced the upregulation of Caspase-8 and p38 via modulating their common target miR-671. However, there have been rare studies elucidating the interactions between lncRNAs/circRNAs and miRNAs in the field of Th2 differentiation in AR.

Here in the current study, the differential expressions of several circRNAs and lncRNAs were compared in nasal mucosa samples of AR patients and mice with experimentally induced AR as compared to healthy controls. The results showed that the highly expressed circRNA (named as CircHIPK3) and lncRNA (named as LncGAS5) promoted Th2 differentiation of ovalbumin- (OVA-) induced CD4^+^ T cells and aggravated nasal symptoms of AR mice. Further experiments indicated that CircHIPK3 and LncGAS5 induced the upregulation of GATA-3 via the common target miR-495. Meanwhile, the intranasal administration of CircHIPK3/LncGAS5-knockdown lentivirus reduced nasal symptoms of AR-induced mice. Our findings provide a novel insight into the interaction among lncRNAs, circRNAs, and miRNAs in AR pathogenesis.

## Materials and methods

### Ethical statement

This study was approved by the Institute Research Medical Ethics Committee of The First Affiliated Hospital of Zhengzhou University. Written informed consents were obtained from all the patients involved and the consent to publish was obtained. All animal experiments were complied with the ARRIVE guidelines, and were carried out in accordance with the U.S. Public Health Service Policy on Humane Care and Use of Laboratory Animals.

### Clinical nasal mucosa samples

Nasal mucosa samples were scraped from the surface of the inferior nasal turbinate of AR patients (*n* = 10) and healthy individuals (*n* = 10) using a plastic curette. The diagnosis of AR was confirmed by otolaryngologists based on the criterion of a typical history of AR over 2 years, with the clinical manifestations, including running nose, positive skin prick test against specific antigens, and serum specific IgE levels greater than 0.3 IU/ml. Individuals who have sinusitis were excluded. The venous blood of AR patients was also collected.

### Animals and groups

Male BALB/c mice aging from 6 to 8 weeks old (weighing 18–21 g) were bought from Shanghai Lab Animal Research Center (Shanghai, China). A total of 48 mice were randomly divided into 8 groups (*n* = 6 in each group): (i) Control + lenti-GFP (normal mice, treated with control lentivirus Lenti-GFP); (ii) AR + lenti-GFP (AR-induced, treated with control lentivirus Lenti-GFP); (iii) AR + lenti-siCircHIPK3 (AR-induced, treated with lentivirus si-CircHIPK3); (iv) AR + lenti-siLncGAS5 (AR-induced, treated with lentivirus si-LncGAS5); (v) AR + lenti-siCircHIPK3 + miR-495 inhibitor (AR-induced, treated with lentivirus si-CircHIPK3 and miR-495 inhibitor); (vi) AR + lenti-siLncGAS5 + miR-495 inhibitor (AR-induced, treated with lentivirus si-LncGAS5 and miR-495 inhibitor); (vii) AR + lenti-siCircHIPK3 + lenti-GATA-3 (AR-induced, treated with lentivirus si-CircHIPK3 and lentivirus GATA-3); (viii) AR + lenti-siLncGAS5 + lenti-GATA-3 (AR-induced, treated with lentivirus si-LncGAS5 and lentivirus GATA-3). All the mice were kept in the animal center of The First Affiliated Hospital of Zhengzhou University with enough food and water under standard conditions. The animal chosen and group-dividing were random and the investigator was blinded to the group allocation.

### Induction of AR in mice

The mouse AR model was established as previously described^13^. Briefly, mice were intraperitoneally injected with ovalbumin (OVA; Sigma-Aldrich, St Louis, MO, USA) (100 μg OVA and 1 mg aluminum hydroxide dissolved in 300 μl saline) on days 0, 7, and 14 (OVA sensitization). Then they were intranasally administered with OVA (400 μg OVA dissolved in 40 μl saline) at a dose of 20 μl per nostril per day from days 21 to 35 (OVA intranasal challenge). The same volume of saline was used intraperitoneally and intranasally in the control group.

### Quantitative real-time PCR (qRT-PCR)

The TRIzol reagent (Thermo Fisher Scientific, Waltham, MA, USA) was used to extract total RNA from tissues and cells, then the cDNA synthesis was done by reverse transcription kit (Takara, Dalian, Liaoning, China) for circRNA, lncRNA, and mRNA, and RiboBio reverse transcription kit for miRNA (Guangzhou, Guangdong, China). The FastStart UniveGDMl SYBR Green Master (Roche, Basel, Switzerland) was used to quantify the expression of genes. The relative expression of circRNA, mRNA and miRNA were calculated by 2^−ΔΔCt^. GAPDH was used as the internal control for mRNA, circRNA, and lncRNA, and U6 was used as the internal control for miRNA.

### Western blot analysis

Cell pellets were lysed with radioimmunoprecipitation assay (RIPA) buffer (Thermo Fisher Scientific) containing phosphatase inhibitor and proteinase cocktails (Sigma-Aldrich, St Louis, MO, USA). Then the resultant proteins were electrophoresed and transferred to a polyvinylidene difluoride (PVDF) membrane (Bio-Rad, Hercules, CA, USA). Membranes were blocked with 5% fetal bovine serum (FBS) for 1 h, followed by the incubation with primary antibodies at 4 °C overnight. The secondary antibody was added and developed with an ECL western blotting substrate (Pierce, Rockford, IL, USA). Primary antibodies used in the study were anti-GATA-3 (ab199428, 1/1000, Abcam, Cambridge, UK), anti-GAPDH (ab8245, 1/500, Abcam), and anti-β-actin (ab8226, 1/500, Abcam).

### Preparation and administration of lentiviral vectors

The vector used to construct the lentiviral vectors was pLV-enhanced GFP (EGFP)-N (lentivirus gene overexpression vector) and pSIH1-H1-copGFP (lentivirus gene silencing vector of shRNA fluorescent expression). The lentiviral vectors of lenti-CircHIPK3, lenti-LncGAS5, lenti-GATA-3, sh-CircHIPK3 (lenti-siCircHIPK3), and sh-LncGAS5 (lenti-siLncGAS5) were constructed by GenePharma Technology (Shanghai, China). Lentiviral packaging was performed using 293T cells. The cells were cultured in RPMI 1640 complete medium containing 10% FBS and sub-cultured every other day. The virus was collected and CD4^+^ T cells were classified into the following groups according to different transfections: OVA + lenti-GFP (transfected with empty vector), OVA + lenti-CircHIPK3 (transfected with CircHIPK3 overexpression lentiviral vector), OVA + lenti-CircHIPK3 (transfected with LncGAS5 overexpression lentiviral vector) or OVA + lenti-GATA-3 (transfected with GATA-3 overexpression lentiviral vector). For the administration of lentiviral vectors, mice were intranasally administered with 2 × 10^6^ IFUs of the lentiviral vectors or empty lentiviral vectors 2 days before day 0^14^.

### Assessment for nasal symptoms

Nasal allergy-like symptoms were measured by counting the numbers of sneezing and nasal rubbing movements after AR induction. Mice were placed into a plastic animal cage (35 × 20 × 30 cm, one animal/cage) and the number of sneezes and nasal rubbing movements for 10 min was counted.

### Measurement of IgE and IL-4

Nasal mucosa and blood were collected from the nostril and the orbital venous plexus 24 h after the final AR induction. The blood samples were centrifuged at 1000× rpm for 10 min, and both nasal mucosa and blood samples were stored at −80 °C until use. Total serum/mucosa IgE/IL-4 was determined using a Mouse IgE/IL-4 ELISA Kit (Thermo Fisher Scientific, Waltham, MA, USA).

### Isolation of CD4^+^ T cells and induction of Th2 differentiation

To obtain human CD4^+^ T cells, the peripheral blood of AR patients was collected. CD4^+^ T cells were isolated from peripheral blood mononuclear cells (PBMCs) by the magnetic-activated cell sorting (MACS). To obtain mouse CD4^+^ T cells, spleen tissues were collected from healthy mice, and CD4^+^ T cells were isolated from spleen tissues as described above. The induction of Th2 differentiation was performed by incubating CD4^+^ T cells with anti-CD3 (3 μg/ml), and anti-CD28 (5 μg/ml) for 7 days. OVA was simultaneously added to induce the inflammatory response. The percentage of Th2 cells was detected using flow cytometry.

### Cell transfection

To evaluate the regulation of CircHIPK3/LncGAS5 on miR-495, CD4^+^ T cells were transfected with small interfering RNAs (siRNAs) against CircHIPK3 (si-CircHIPK3) or LncGAS5 (si-LncGAS5) and the negative control (NC) siRNAs (si-control), as well as miR-495 mimic/inhibitor and their control Pre-NC/NC using Lipofectamine 2000 (Thermo Fisher Scientific, Waltham, MA, USA) according to the manufacturer’s instructions. The siRNAs of CircHIPK3, LncGAS5 and GATA was constructed by Ribobio (Guangzhou, China). The sequences were shown as follows: si-CircHIPK3: 5′-CUACAGGUAUGGCCUCACA-3′; si-LncGAS5: 5′-GCGAGCGCAATGTAAGCAA-3′; si-GATA-3: 5′-AAGAUGAGAAAGAGUGCCUCA-3′.

### Luciferase reporter assay

Cells were transfected with Renilla and firefly luciferase plasmids carrying mutant (MUT) or wild type (WT) CircHIPK3, LncGAS5, or 3′-UTR of GATA-3 at 80–90% confluence together with miR-495 mimic or inhibitor. The passive lysis buffer (PLB) was added to the samples after washing with PBS, and cells were incubated for 15 min at room temperature. After the centrifugation at 10,000 rpm for 5 min at 4 °C, the supernatants were removed. A total of 20 μl samples were transferred to a 96-well plate and mixed with 100 μl Dual-Glo Luciferase Assay System (Promega, Madison, WI, USA). The relative luciferase activity was measured.

### RNA antisense purification (RAP)

The RAP assay was done by using the RAP kit (BersinBio, Guangzhou, Guangdong, China) as reported^12^. In brief, cells were lysed with 1 ml lysis buffer and fully homogenized with a 0.4 mm syringe. The biotinylated antisense probe of lncGAS5 (0.2 nmol) was added to the lncRNA-RAP system. The biotinylated antisense probe of CircHIPK3 (0.2 nmol) was added to the circRNA-RAP system. The probes were denatured for 10 min at 65 °C and hybrided for 2 h at room temperature, followed by adding 200 μl streptavidin-coated magnetic beads. After removing the non-specifically bound RNAs, qRT-PCR was done to analyze the expression of miR-495 in the pulled down complex.

### RNA pull-down assay

The miR-495 was biotin-labeled with the Biotin RNA labeling mix (Sigma-Aldrich, St Louis, MO, USA). Positive (Input), negative control (Biotin NC), and biotinylated miR-495 were mixed and incubated with 293T cell lysates. After adding the magnetic beads, the samples were incubated at room temperature for 2 h. Then, the beads were washed, and the relative expression of CircHIPK3 and LncGAS5 were examined using qRT-PCR.

### Fluorescence in situ hybridization (FISH)

Cells were fixed with 4% paraformaldehyde for 15 min at room temperature, then they were washed twice with 0.1% diethylpyrocarbonate solution followed by the treatment with 0.5% Triton X-100 (Solarbio Life Sciences, Beijing, China) for 5 min. The samples were dehydrated in a graded series of alcohol and air-dried. The probe hybridization solution was added to the samples, then the samples were mounted, denatured for 3 min at 73 °C before hybridizing in a dark and humid environment for 12–16 h at 37 °C with the Cy5-labeled lncRNA probe, 6-carboxyfluorescein-labeled circRNA probe, and Cy3-labeled miRNA probe (all from BersinBio). After washing the samples for three times with a pre-heated (43 °C) solution containing 2× saline sodium citrate (SSC) and 50% formamide, the samples were then washed twice with 2× SSC (37 °C). The samples were counterstained with 4′, 6-diamidino-2-phenylindole, followed by being mounted with fluorescence mounting medium. Then they were observed with a microscope.

### Statistical analysis

Each experiment was repeated more than three times. The data are represented as the mean ± standard deviation (SD). We used Student’s *t* test or one‐way analysis of variance (one‐way ANOVA) followed by LSD post hoc test to compare the differences between two groups or more than two groups, respectively. The SPSS (SPSS 19.0; SPSS Inc, Chicago, IL, USA) was used to perform the statistical analysis. The *P* value < 0.05 was considered as statistically significant.

## Results

### Identification of circRNAs and lncRNAs differentially expressed in AR nasal mucosa

**Fig. 1:**
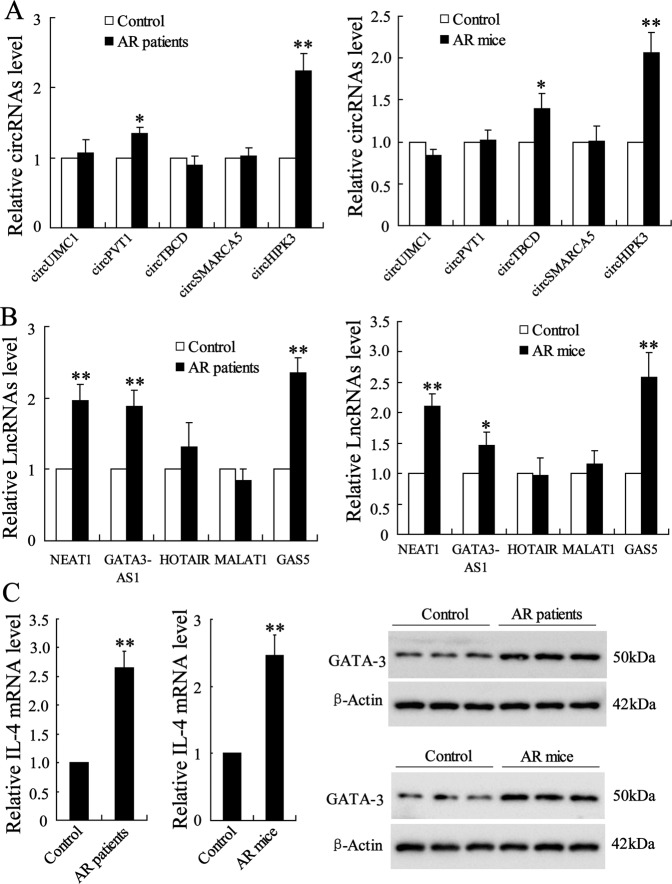
Identification of circRNAs and lncRNAs differentially expressed in AR nasal mucosa. Clinical samples of nasal mucosa were scraped from the surface of the inferior nasal turbinate of AR patients (*n* = 10) and healthy individuals (*n* = 10) using a plastic curette. Mouse AR was induced by OVA sensitization and OVA intranasal challenge. The murine samples of nasal mucosa were collected from AR-induced mice (*n* = 6) and control mice (*n* = 6). **a**, **b** The expressions of five circRNAs and five lncRNAs in mucosa samples were detected using qRT-PCR. **c** The expression of IL-4 and GATA-3 in mucosa samples was detected using qRT-PCR and western blot analysis, respectively. *p < 0.05, **p < 0.01 vs Control.

Clinical samples of nasal mucosa were collected from AR patients and healthy patients, while the murine samples of nasal mucosa were collected from AR-induced mice and control mice. The expressions of several circRNAs which were reported to be related to the immunity regulation^15^, including circUIMC1, circPVT1, circTBCD, circSMARCA5, and circHIPK3, were detected in mucosa samples. The result showed that only CircHIPK3 was highly expressed both in clinical and murine AR mucosa in comparison with the control samples (Fig. 1a, *p* < 0.01). Meanwhile, the expression of several lncRNAs which were reported to be related to CD4^+^ T cell differentiation^16^, including NEAT1, GATA3-AS1, HOTAIR, MALAT1, and GAS5, were detected in mucosa samples. The result showed that NEAT1 (both *p* < 0.01), GATA3-AS1 (*p* < 0.01 in clinical samples, *p* < 0.05 in murine samples), and GAS5 (both *p* < 0.01) were highly expressed in clinical and murine AR mucosa in comparison with the control samples (Fig. 1b). Since the effect of NEAT1 and GATA3-AS1 on Th2 differentiation has been previously explored^17,18^, LncGAS5 was chosen for further investigations in this study. The enhancement of IL-4 and GATA-3 expressions was also confirmed in AR mucosa compared with the control (Fig. 1c), suggesting the promotion of Th2 differentiation in AR pathogenesis. Taken together, CircHIPK3 and LncGAS5 were significantly upregulated in AR mucosa, and their effects on AR symptoms and Th2 differentiation were investigated in the next experiments.

### Knockdown of CircHIPK3 or LncGAS5 alleviates nasal symptoms of AR mice

**Fig. 2:**
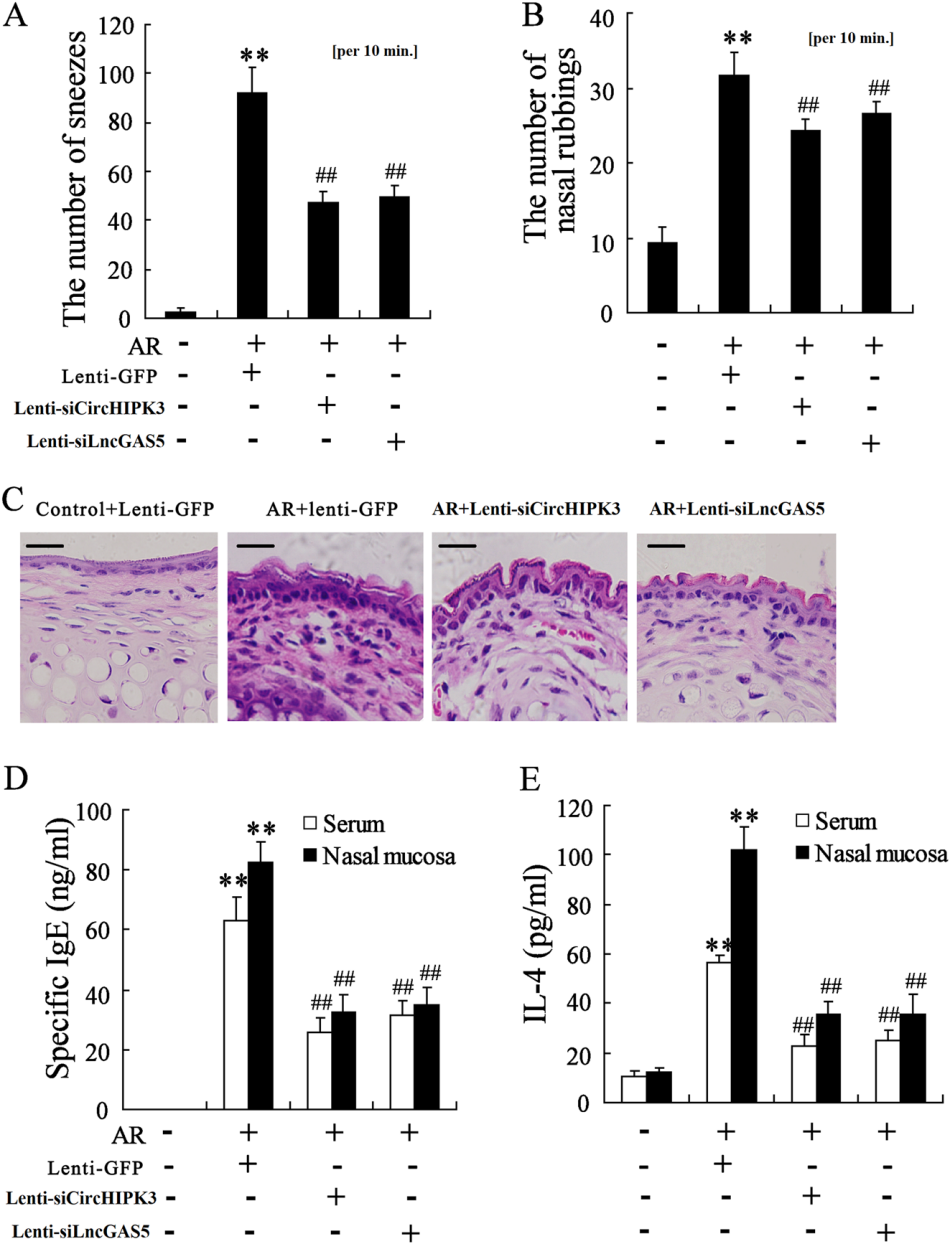
Knockdown of CircHIPK3 or LncGAS5 alleviates nasal symptoms of AR mice. AR mice were intranasally treated with CircHIPK3 knockdown lentivirus (lenti-siCircHIPK3) or LncGAS5-knockdown lentivirus (lenti-siLncGAS5) (*n* = 6 in each group). **a**, **b** The number of sneezes and nasal rubbings. **c** H&E staining of the nasal mucosa (Scale bar = 10 μm). **d**, **e** Levels of IgE and IL-4 in serum and nasal mucosa were detected using ELISA. **p < 0.01 vs Control + lenti-GFP. ^##^*p* < 0.01 vs AR + lenti-GFP.

AR mice were intranasally treated with CircHIPK3 or LncGAS5-knockdown lentivirus to investigate the effect of CircHIPK3/LncGAS5 knockdown on AR symptoms in vivo. The results indicated that both treatments markedly reduced the nasal symptoms of AR mice, including the number of sneezes and nasal rubbings (Fig. 2a, b, *p* < 0.01). The H&E staining showed that the inflammatory response of nasal mucosa was decreased with the reduced infiltration of inflammatory cells after both treatments (Fig. 2c). The downregulated levels of IgE and IL-4 in serum and nasal mucosa in the treatment group also suggested the anti-allergic effect of CircHIPK3/LncGAS5 knockdown (Fig. 2d, e). These data indicated that the knockdown of CircHIPK3 or LncGAS5 alleviated nasal symptoms of AR mice.

### Overexpression of CircHIPK3 or LncGAS5 promotes Th2 differentiation of OVA-induced CD4^+^ T cells

**Fig. 3:**
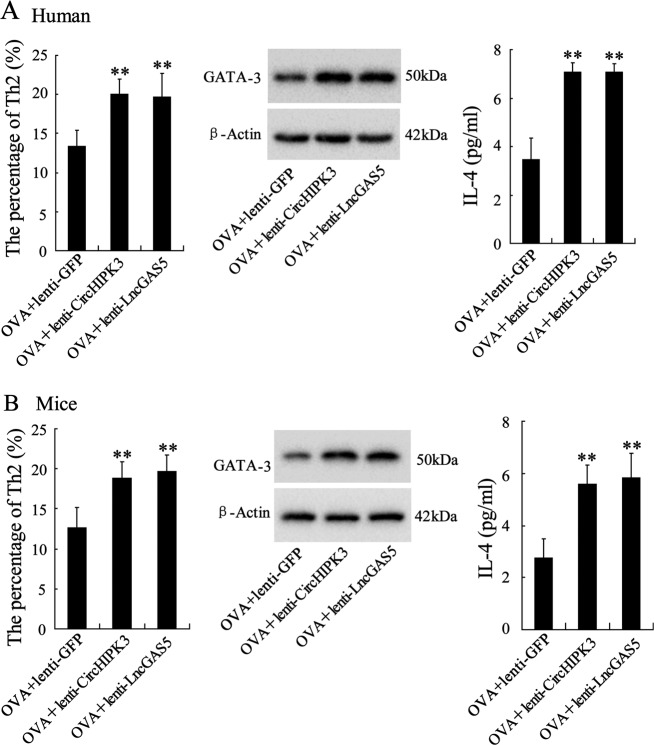
Overexpression of CircHIPK3 or LncGAS5 promotes Th2 differentiation of OVA-induced CD4^+^ T cells. CD4^+^ T cells, isolated from PBMCs of AR patients or spleen tissues of mice, were induced by OVA and Th2 inducing agents for 7 days after the transfection of CircHIPK3 lentivirus (lenti-CircHIPK3) or LncGAS5 lentivirus (lenti-LncGAS5). **a**, **b** The percentage of Th2 cells, the protein level of GATA-3, and the supernatant level of IL-4 were detected using flow cytometry, western blot analysis, and ELISA, respectively. **p < 0.01 vs OVA + lenti-GFP.

After overexpressing CircHIPK3 or LncGAS5, CD4^+^ T cells were induced by OVA to trigger allergic responses followed by the induction of Th2 differentiation to investigate the effect of CircHIPK3/LncGAS5 knockdown on Th2 differentiation in vitro. In human isolated CD4^+^ T cells, the overexpression of CircHIPK3 or LncGAS5 significantly increased the percentage of Th2 cells, the protein level of GATA-3, and the supernatant level of IL-4 (Fig. 3a). Similarly, in mouse isolated CD4^+^ T cells, the overexpression of CircHIPK3 or LncGAS5 significantly increased the percentage of Th2 cells, the protein level of GATA-3, and the supernatant level of IL-4 (Fig. 3b). These findings indicated that both CircHIPK3 and LncGAS5 promoted Th2 differentiation of OVA-induced CD4^+^ T cells.

### CircHIPK3 and LncGAS5 interact directly with miR-495 and regulate its expression

**Fig. 4:**
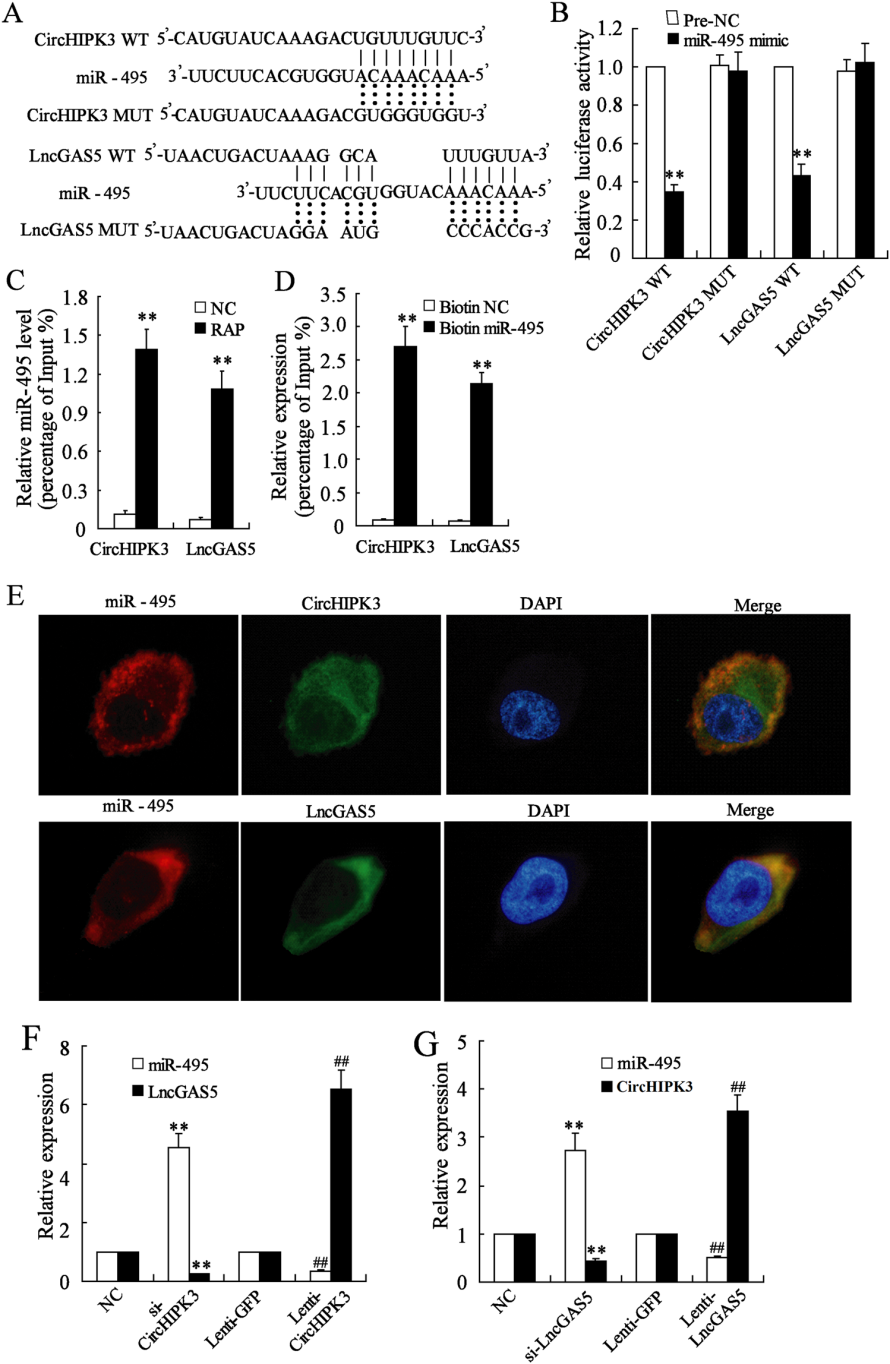
CircHIPK3 and LncGAS5 interact directly with miR-495 and regulate its expression. **a** The predicted binding sequences and the artificially mutated sequences between CircHIPK3/LncGAS5 and miR-495. **b** The relative luciferase activity after the co-transfection with miR-495 mimic and the CircHIPK3/LncGAS5 mutant/wild type (MUT/WT) reporter vector. **c** The relative miR-495 level in the complex which was pulled down by CircHIPK3 or LncGAS5 probe using the RAP assay. **d** Relative expression of CircHIPK3 and LncGAS5 in the complex which was pulled down by biotin-labeled miR-495 using the RNA pull-down assay. **e** The cellular distribution of CircHIPK3, LncGAS5, and miR-495 in OVA and Th2 differentiation-induced CD4^+^ T cells using the FISH assay. **f** Relative expression of LncGAS5 and miR-495 after CircHIPK3 knockdown or overexpression. **g** Relative expression of CircHIPK3 and miR-495 after LncGAS5 knockdown or overexpression. **p < 0.01 vs negative controls (pre-NC, Biotin NC, or NC). ^##^*p* < 0.01 vs lentivirus control (Lenti-GFP).

To clarify the mechanism underlying the effects of CircHIPK3 and LncGAS5 on Th2 differentiation in AR, we used the online database RNA Association Interaction Database (RAID v2.0) and CircBase (circrna.org) to predict the interactions between CircHIPK3/LncGAS5 and miRNAs. We found that there were some sequences in miR-495 which could potentially bind with CircHIPK3 and LncGAS5 (Fig. 4a). The luciferase reporter assay showed that miR-495 overexpression did not change the luciferase activity after mutating the predicted sequences in the reporter vector (the CircHIPK3 MUT group), while it markedly reduced the luciferase activity in the WT group, and the similar results were also observed in LncGAS5 (Fig. 4b). The RAP assay showed that miR-495 was accumulated in the complex which was pulled down by CircHIPK3 or LncGAS5 probe (Fig. 4c). Meanwhile, the RNA pull-down assay showed that CircHIPK3 and LncGAS5 were enriched in the complex which was pulled down by biotin-labeled miR-495 (Fig. 4d). The database also predicted the potential binding between CircHIPK3/LncGAS5 and miR-338-3p (Fig. S1A). However, miR-338-3p overexpression did not change the luciferase activity in the WT group (Fig. S1B), and miR-338-3p was not markedly accumulated in the complex which was pulled down by CircHIPK3 or LncGAS5 probe (Fig. S1C). These data indicated that CircHIPK3 and LncGAS5 could interact directly and specifically with miR-495.

To visualize the cellular distribution of these molecules, FISH assay was done in OVA and Th2 differentiation-induced CD4^+^ T cells. CircHIPK3 and miR-495, as well as LncGAS5 and miR-495, were expressed in the cytoplasm in the same pattern, suggesting that they interacted via post-transcriptional mechanisms (Fig. 4e). CircHIPK3 overexpression inhibited miR-495 expression and promoted LncGAS5 expression, while CircHIPK3 knockdown promoted miR-495 expression and inhibited LncGAS5 expression (Fig. 4f). Similarly, LncGAS5 overexpression inhibited miR-495 expression and promoted CircHIPK3 expression, while LncGAS5 knockdown promoted miR-495 expression and inhibited CircHIPK3 expression (Fig. 4g). These findings indicated a positive regulation between CircHIPK3 and LncGAS5 and a negative regulation between CircHIPK3/LncGAS5 and miR-495.

### CircHIPK3 and LncGAS5 increase GATA-3 expression via downregulating miR-495

**Fig. 5:**
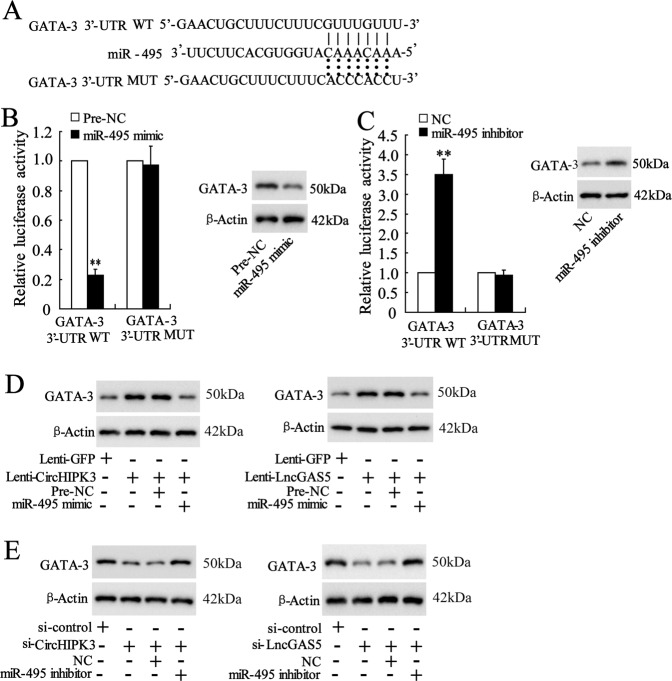
CircHIPK3 and LncGAS5 increase GATA-3 expression via downregulating miR-495. **a** The predicted binding sequences and the artificially mutated sequences between miR-495 and the 3′-untranslated region (UTR) of GATA-3. **b** The relative luciferase activity after the co-transfection with miR-495 mimic and the GATA-3 3′-UTR mutant/wild type (MUT/WT) reporter vector. **c** The relative luciferase activity after the co-transfection with miR-495 inhibitor and the GATA-3 3′-UTR mutant/wild type (MUT/WT) reporter vector. **d** CD4^+^ T cells were transfected with Lenti-CircHIPK3/Lenti-LncGAS5 or co-transfected with miR-495 mimic followed by the induction of OVA and Th2 differentiation. The GATA-3 protein level was detected using western blot analysis. **e** CD4^+^ T cells were also transfected with si-CircHIPK3/si-LncGAS5 or co-transfected with miR-495 inhibitor followed by the induction of OVA and Th2 differentiation. The GATA-3 protein level was detected using western blot analysis. **p < 0.01 vs negative controls (pre-NC or NC).

The database RAID also predicted the potential binding between miR-495 and the 3′-untranslated region (UTR) of GATA-3 (Fig. 5a). The overexpression of miR-495 did not change the luciferase activity after mutating the predicted sequences in the reporter vector (the GATA-3 MUT group), while it markedly reduced the luciferase activity in the WT group, and miR-495 overexpression reduced GATA-3 protein expression (Fig. 5b). On the other hand, the knockdown of miR-495 did not change the luciferase activity after mutating the predicted sequences in the reporter vector (the GATA-3 MUT group), while it markedly enhanced the luciferase activity in the WT group, and miR-495 knockdown raised GATA-3 protein expression (Fig. 5c). These data suggested that GATA-3 was a downstream target of miR-495. To elucidate the relationship among CircHIPK3/LncGAS5, miR-495, and GATA-3, CD4^+^ T cells were transfected with Lenti-CircHIPK3/Lenti-LncGAS5 or co-transfected with miR-495 mimic followed by the induction of OVA and Th2 differentiation. The result indicated that the co-transfection reduced the enhancement of GATA-3 protein level which was raised by CircHIPK3/LncGAS5 overexpression (Fig. 5d). They were also transfected with si-CircHIPK3/si-LncGAS5 or co-transfected with miR-495 inhibitor before the induction. The result indicated that the co-transfection restored the decrease of GATA-3 protein level which was defeated by CircHIPK3/LncGAS5 knockdown (Fig. 5e). Taken together, these data suggested that CircHIPK3 and LncGAS5 increased GATA-3 expression via downregulating miR-495.

### CircHIPK3 and LncGAS5 promote Th2 differentiation of OVA-induced CD4^+^ T cells via miR-495/GATA-3 pathway

**Fig. 6:**
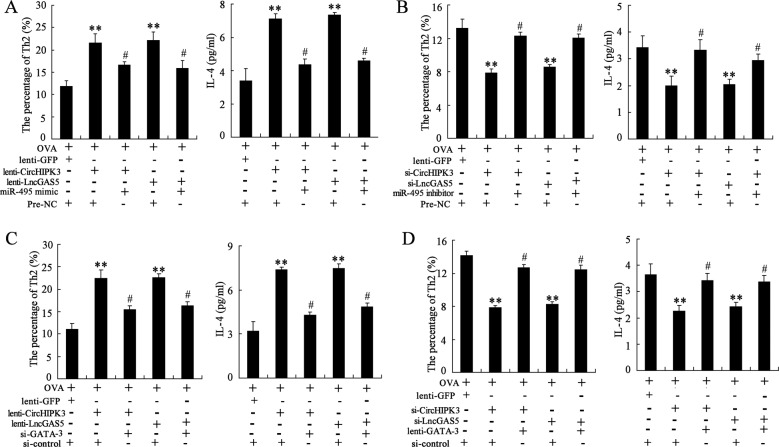
CircHIPK3 and LncGAS5 promote Th2 differentiation of OVA-induced CD4^+^ T cells via miR-495/GATA-3 pathway. **a** CD4^+^ T cells were induced by OVA and Th2 inducing agents for 7 days after being transfected with Lenti-CircHIPK3/Lenti-LncGAS5 or co-transfected with miR-495 mimic. The percentage of Th2 cells and the supernatant level of IL-4 were detected using flow cytometry, and ELISA, respectively. **b** CD4^+^ T cells were induced by OVA and Th2 inducing agents for 7 days after being transfected with si-CircHIPK3/si-LncGAS5 or co-transfected with miR-495 inhibitor. The percentage of Th2 cells and the supernatant level of IL-4 were detected. **c** CD4^+^ T cells were induced by OVA and Th2 inducing agents for 7 days after being transfected with Lenti-CircHIPK3/Lenti-LncGAS5 or co-transfected with si-GATA-3. The percentage of Th2 cells and the supernatant level of IL-4 were detected. **d** CD4^+^ T cells were induced by OVA and Th2 inducing agents for 7 days after being transfected with si-CircHIPK3/si-LncGAS5 or co-transfected with Lenti-GATA-3. The percentage of Th2 cells and the supernatant level of IL-4 were detected. **p < 0.01 vs OVA + lenti-GFP + Pre-NC/si-control. ^#^*p* < 0.05 vs OVA + Lenti-CircHIPK3/Lenti-LncGAS5 + Pre-NC or OVA + si-CircHIPK3/si-LncGAS5 + si-control.

After transfection, CD4^+^ T cells were induced by OVA and Th2 inducing agents for 7 days. The overexpression of CircHIPK3/LncGAS5 increased the percentage of Th2 cells and the IL-4 level, and such response was negated by the co-transfection with miR-495 mimic (Fig. 6a). On contrast, the knockdown of CircHIPK3/LncGAS5 reduced the percentage of Th2 cells and the IL-4 level, and such response was negated by the co-transfection with miR-495 inhibitor (Fig. 6b). On the other hand, the promotion of CircHIPK3/LncGAS5 overexpression on Th2 differentiation was negated by the co-transfection of si-GATA-3 (Fig. 6c), while the inhibition of CircHIPK3/LncGAS5 knockdown on Th2 differentiation was negated by the co-transfection of lenti-GATA-3 (Fig. 6d). Our findings indicated that CircHIPK3 and LncGAS5 promoted Th2 differentiation of OVA-induced CD4^+^ T cells via decreasing miR-495 expression and subsequently increasing GATA-3 expression.

### Knockdown of CircHIPK3 or LncGAS5 alleviates nasal symptoms of AR mice via miR-495/GATA-3 pathway

**Fig. 7:**
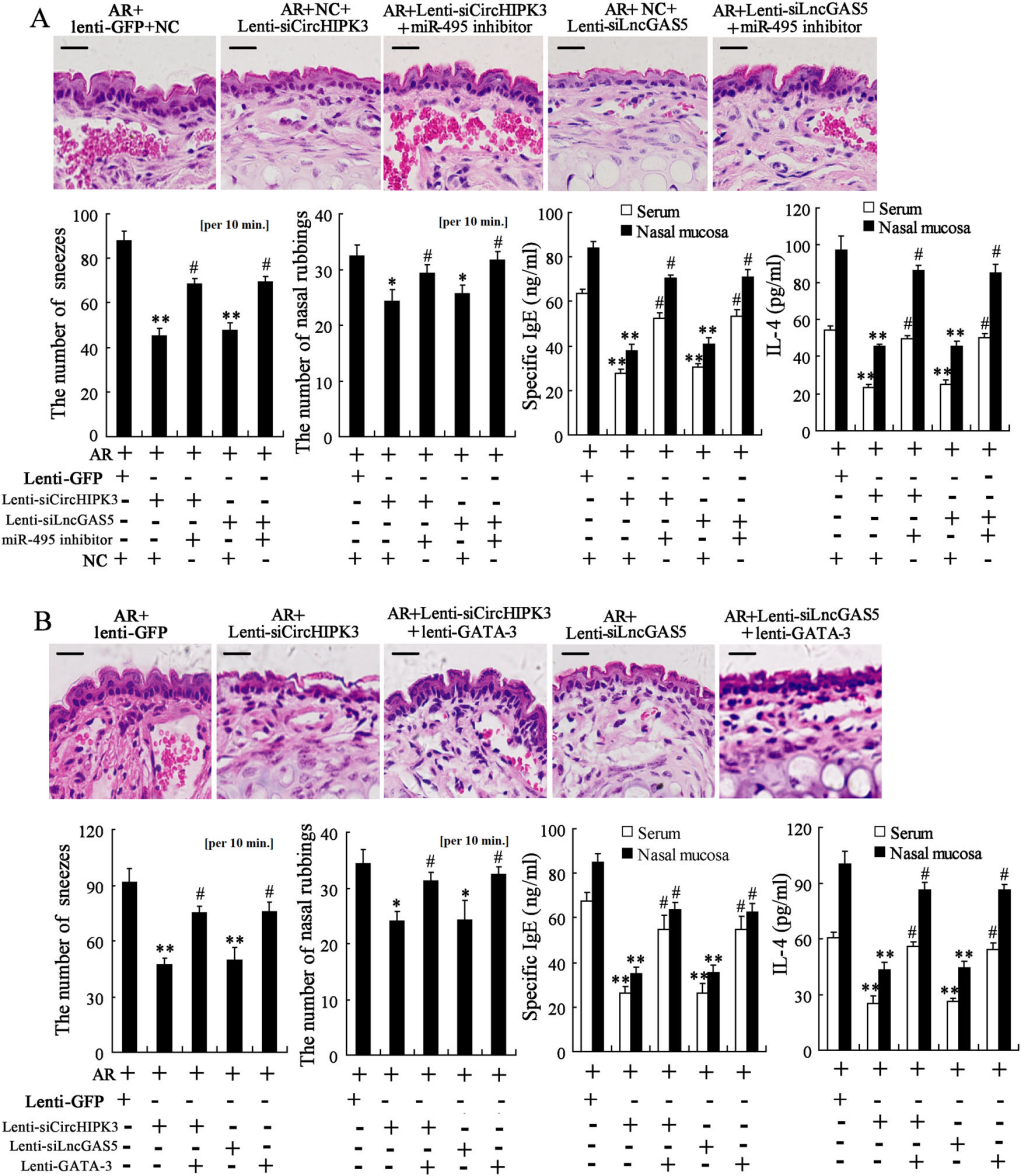
Knockdown of CircHIPK3 or LncGAS5 alleviates nasal symptoms of AR mice via miR-495/GATA-3 pathway. **a** AR-induced mice were treated intranasally with CircHIPK3/LncGAS5-knockdown lentivirus, or together with miR-495 inhibitor (*n* = 6 in each group). The H&E staining of nasal mucosa (Scale bar = 10 μm), the number of sneezes and nasal rubbings, and the levels of IgE and IL-4 in serum and nasal mucosa were shown. **b** AR-induced mice were treated intranasally with CircHIPK3/LncGAS5-knockdown lentivirus, or together with GATA-3-overexpressing lentivirus (*n* = 6 in each group). The H&E staining of the nasal mucosa (Scale bar = 10 μm), the number of sneezes and nasal rubbings, and the levels of IgE and IL-4 in serum and nasal mucosa were shown. *p < 0.05, **p < 0.01 vs AR + lenti-GFP + NC or AR + lenti-GFP. ^#^*p* < 0.05 vs AR + Lenti-siCircHIPK3/Lenti-siLncGAS5 + NC or AR + Lenti-siCircHIPK3/Lenti-siLncGAS5.

AR-induced mice were treated intranasally with CircHIPK3/LncGAS5-knockdown lentivirus, or together with miR-495 inhibitor. The H&E staining results showed that the administration of CircHIPK3/LncGAS5-knockdown lentivirus decreased the inflammatory response of nasal mucosa as the infiltration of inflammatory cells and red blood cells were reduced, while such response was negated by the administration of miR-495 inhibitor (Fig. 7a). The administration of CircHIPK3/LncGAS5-knockdown lentivirus also alleviated nasal symptoms and lowered the levels of IgE and IL-4 in serum and nasal mucosa, while such therapeutic effects were negated by the administration of miR-495 inhibitor (Fig. 7a).

On the other hand, AR-induced mice were treated intranasally with CircHIPK3/LncGAS5-knockdown lentivirus, or together with GATA-3-overexpressing lentivirus. The H&E staining results showed that the administration of GATA-3-overexpressing lentivirus aggravated the inflammatory response of nasal mucosa which was alleviated by CircHIPK3/LncGAS5-knockdown (Fig. 7b). The administration of GATA-3-overexpressing lentivirus also aggravated the nasal symptoms and restored the levels of IgE and IL-4 in serum and nasal mucosa (Fig. 7b). In summary, these findings demonstrated that the therapeutic effect of CircHIPK3/LncGAS5 knockdown was mediated by miR-495/GATA-3 pathway.

## Discussion

Th2 cells are responsible for the inflammation. On the one hand, Th2 cells produce pro-inflammatory cytokines, such as IL-4, IL-13, and IL-5, and subsequently induce the effector cells like eosinophils. On the other hand, Th2 cells induce B lymphocytes into plasma cells, and subsequently promote the release of IgE^19^. The significantly increased polarization to Th2 cells of CD4^+^ T cells was observed in AR^3^. Therefore, identifying the mechanisms underlying Th2 differentiation contributes to the treatment of AR. In the current study, we explored the interactions among three types of ncRNAs, including circRNAs, lncRNAs, and miRNAs, and found that CircHIPK3 and LncGAS5 induced the GATA-3 expression via modulating the common target miR-495, thus promoting Th2 differentiation and aggravating AR.

CircRNAs are a novel subset of ncRNAs whose functions were investigated in recent years^20^. The covalently closed continuous loop structure makes them to be highly stable, conserved, and abundant across different species^21^. Such characteristics of circRNAs give them more attention around the fields, as an increasing number of researchers focused on the regulatory functions in various diseases, including cancers, cardiovascular diseases, and neurological diseases^11,22,23^. However, the effect of circRNAs on AR pathogenesis is rarely reported. In our study, the expressions of several immune-related circRNAs were detected in nasal mucosa samples from AR patients and AR-induced mice, and the dysregulation of CircHIPK3 was identified. The functional experiments confirmed the pro-Th2 differentiation function of CircHIPK3 in vivo and in vitro. Therefore, the regulatory mechanism of CircHIPK3 in Th2 differentiation was further investigated. As reported, the main function of circRNAs is post-transcriptional regulation. Firstly, circRNAs can competitively inhibit the transcriptional regulation of miRNAs by directly binding with them, which is also known as the ceRNA mechanism^24^. In the previous study, CircHIPK3 was reported to regulate lung fibroblast-to-myofibroblast transition by functioning as a ceRNA^25^. Consistent with their study, we found that CircHIPK3 could directly bind with miR-495, thus negating the transcriptional inhibition of miR-495 on GATA-3 mRNA. Meanwhile, CircHIPK3 could also negatively regulate miR-495 expression, although the specific mechanism deserves further investigations. Secondly, circRNAs can directly bind with proteins, such as the miRNA effector protein, AGO^26^. Du et al.^27^ also reported that circFoxo3 blocked the progression of the cell cycle by binding to the cell cycle protein CDK2 and p21. Thirdly, circRNAs can even bind with base pairs of RNAs, thus producing large RNA-protein complexes^28^. Since the functions of circRNAs are still largely unknown, whether CircHIPK3 has other functions in regulating Th2 differentiation will be explored in our future researches.

LncRNAs are another subset of ncRNAs that have been widely investigated in immune regulation. For instance, lncRNA MAF-4 inhibited Th2 differentiation of CD4^+^ T cells via directly inhibiting MAF protein^29^. LncRNA Ccr2-5′AS, which is a Th2-cell specific lncRNA, was essential for the regulatory circuit in Th2-specific gene expression^30^. In the present study, LncGAS5 was found to be highly expressed in nasal mucosa samples from both AR patients and AR-induced mice. The knockdown of LncGAS5 reduced nasal symptoms of AR mice, while the overexpression of LncGAS5 promoted Th2 differentiation of OVA-induced CD4^+^ T cells. These data suggested that LncGAS5 may play a critical role in Th2 differentiation of AR. Previously, LncGAS5 was firstly discovered from growth arrest murine fibroblasts^31^, and its tumor-suppressing function was also identified in colorectal cancers, cervical cancers, and other cancers^32,33^. A study conducted by Mourtada-Maarabouni et al.^34^ demonstrated that LncGAS5 increased the apoptosis and reduced the rate of progression of T cells through the cell-cycle, indicating the regulatory function of LncGAS5 in T cell fate. Consistent with their study, our findings indicated the regulatory function of LncGAS5 in T cell differentiation, which is mediated by sponging miR-495 and subsequently raising GATA-3 expression.

Although the interactions between circRNAs and miRNAs, as well as lncRNAs and miRNAs, are well-identified, the interactions among these three types of ncRNAs are rarely reported. In a study conducted by Nan et al.^12^, a dysregulated lncRNA lncRpa and a dysregulated circRNA circRar1 were demonstrated to regulate the common target miR-671, therefore affect the neuronal apoptosis in lead-induced neurotoxicity. Inspired by their study, we investigated the relationship among CircHIPK3, LncGAS5, and miR-495 in Th2 differentiation, and found a similar result, referring that CircHIPK3 and LncGAS5 regulate GATA-3 expression via the common target miR-495. Moreover, the result showed that the expression levels of CircHIPK3 and LncGAS5 affected each other, however, the specific mechanisms were still needed to be solved.

In conclusion, our findings indicated that CircHIPK3 and LncGAS5 promoted Th2 differentiation and aggravated AR via modulating the common target miR-495. The intranasal administration of CircHIPK3/LncGAS5-knockdown lentivirus decreased AR symptoms via downregulating GATA-3, providing a potential therapeutic target of treating AR.

## Supplementary information

The relationship between CircHIPK3/LncGAS5 and miR-338-3p.

## Data Availability

The datasets used and/or analyzed during the current study are available from the corresponding author on reasonable request.
