# Supplementary material for: The regulatory network among CircHIPK3, LncGAS5, and miR-495 promotes Th2 differentiation in allergic rhinitis
Source: Cell Death Dis. 2020 Apr 2;11(4):216. doi: 10.1038/s41419-020-2394-3 (PMC7118158; doi:10.1038/s41419-020-2394-3)
Supplement: Supplementary file 1 — The relationship between CircHIPK3/LncGAS5 and miR-338-3p. [file 41419_2020_2394_MOESM1_ESM.docx]

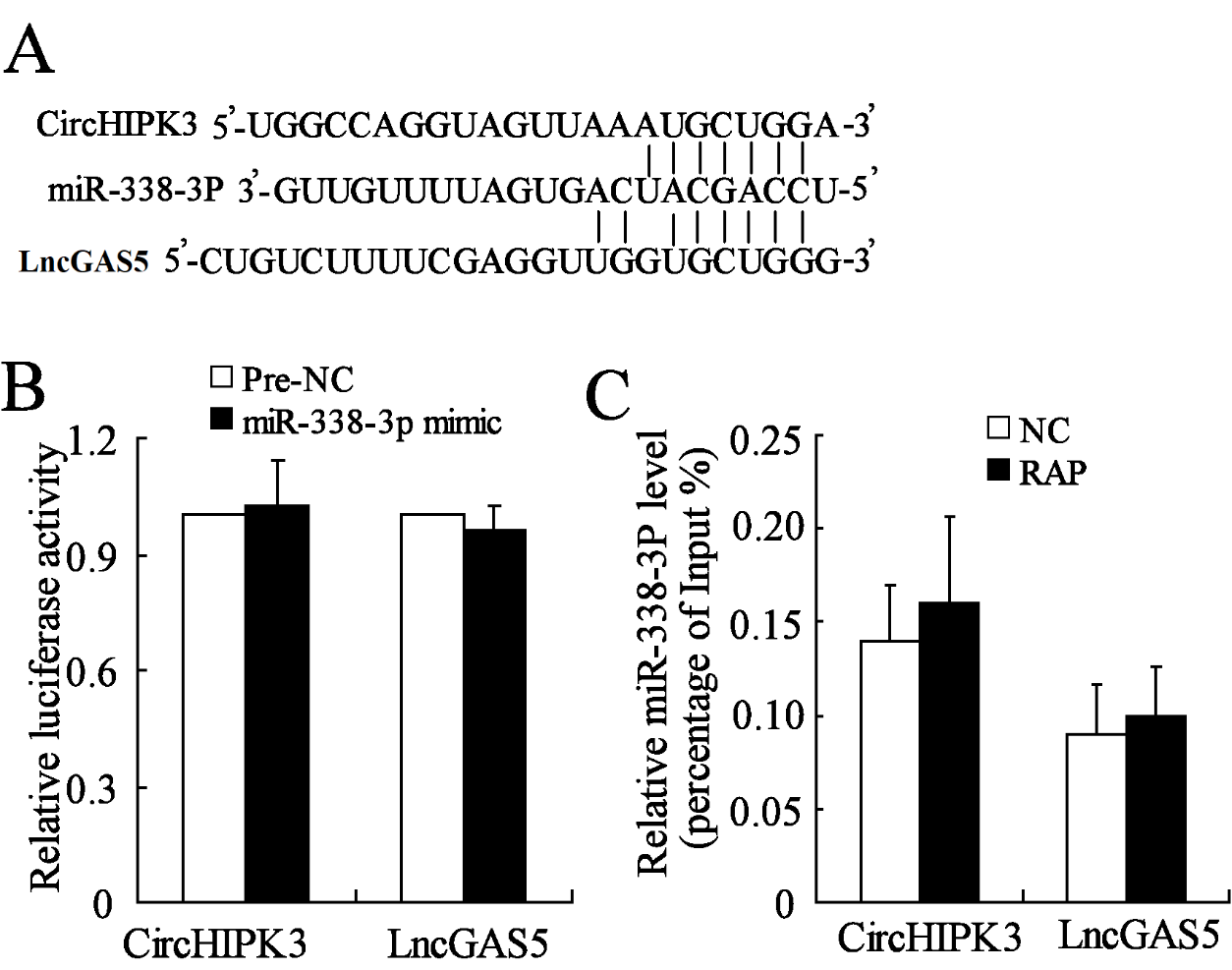


Suppl. Fig. 1 The relationship between CircHIPK3/LncGAS5 and miR-338-3p.

A The predicted binding sequences between CircHIPK3/LncGAS5 and miR-338-3p. B The relative luciferase activity after the co-transfection with miR-338-3p mimic and the CircHIPK3/LncGAS5 wild type reporter vector. C The relative miR-338-3p level in the complex which was pulled down by CircHIPK3 or LncGAS5 probe using the RAP assay.
